# Cadmium toxicokinetics differ between forest and city colonies of the ant *Temnothorax nylanderi* (Formicidae: Myrmicinae) (Foerster, 1850)

**DOI:** 10.1007/s10646-026-03053-4

**Published:** 2026-02-19

**Authors:** Marie Gressler, Angélique Bultelle, Bernard Kaufmann, Mathieu Molet, Claudie Doums

**Affiliations:** 1 Sorbonne Université, UPEC, CNRS, IRD, INRA, Institute of Ecology and Environmental Sciences (IEES-Paris), 4 place Jussieu, 75005, Paris, France; 2 Institut de Systématique, Evolution, Biodiversité (ISYEB), EPHE-PSL, Muséum National D’Histoire Naturelle, CNRS, Sorbonne Université, Université des Antilles, 57 rue Cuvier, CP 50, 75005, Paris, France; 3 EPHE, PSL University, Paris, France; 4 Université Claude Bernard Lyon 1, LEHNA UMR 5023, CNRS, ENTPE, F-69622, Villeurbanne, France

**Keywords:** Urbanisation, Trace metals, Social insects, Bioaccumulation, Assimilation, Elimination

## Abstract

City environments are often contaminated with trace metals, such as cadmium, posing significant risks to organisms. However, how these contaminants accumulate in social groups like ants remains understudied. In this experiment, we compared the temporal kinetics of cadmium accumulation and elimination in colonies of the ant *Temnothorax nylanderi* exposed to the same cadmium concentration but originating from two contrasting habitat types: unpolluted (forest) and polluted (city). Ant colonies were given cadmium-laced food during a contamination phase, followed by uncontaminated food during a decontamination phase, under controlled laboratory conditions. The internal cadmium concentration in ants increased quickly during the contamination phase and reached a plateau. During the decontamination phase, the internal cadmium concentration decreased sharply. Although the global toxicokinetics showed a similar pattern in both habitat types, the plateau was lower in the city than forest habitats. This suggests a more efficient regulation of internal cadmium concentration in colonies living in polluted areas, associated with uptake and not elimination. These findings support the idea that city colonies may have developed enhanced tolerance to cadmium exposure by decreasing their level of contamination for a given contaminated food source. Further studies are needed to investigate the long-term ecological and evolutionary implications of these mechanisms and their relevance for ecosystem pollution monitoring based on ant contamination.

**Supplementary Information:**

The online version contains supplementary material available at 10.1007/s10646-026-03053-4.

## Introduction

Environmental pollution poses a significant threat to both biodiversity and human health, with trace metals being of particular concern due to their persistence and potential for bioaccumulation within food chains (Gall et al. 2015; Ali and Khan 2019; Soliman et al. 2022). Despite regulatory efforts, trace metal pollution remains prevalent in the environment, such as air (Moreno et al. 2011), soil (Foti et al. 2017), and water (Mohiuddin et al. 2010). They continue to harm organisms, even at doses below legal limits (Monchanin et al. 2021). Among the affected organisms, terrestrial soil-dwelling invertebrates are particularly noteworthy, both for their ecological relevance as pollinators, decomposers, contributors to soil aeration, and trophic resources (Stork and Eggleton 1992; Lavelle et al. 2006) and for their role as bioindicators and bioremediators (Stork and Eggleton 1992; Cortet et al. 1999; Lavelle et al. 2006; Khan et al. 2017). Their capacity to accumulate metals (Heikens et al. 2001; Ardestani et al. 2014) further highlights their importance in monitoring pollution. With insects facing widespread declines and conservation efforts lagging, studying the effects of environmental pollutants on insect populations is critical for understanding and mitigating broader ecosystem consequences (van der Sluijs 2020). Trace metals have significant toxicological effects, hindering development and growth (geometrid moth *Epirrita autumnata*: Van Ooik et al. 2007), reducing immunocompetence (ant *Formica aquilonia*: Sorvari et al. 2007; honey bee *Apis mellifera*: Polykretis et al. 2016; bee *Apis cerana cerana*: Li et al. 2024), and impairing cognitive functions (honeybee *A. mellifera*: Monchanin et al. 2024). Exposure to trace metals also impairs locomotion, reproduction, and feeding behaviours in insects, reducing ecosystem services (Eeva et al. 2004; Mogren and Trumble 2010; Jaffe et al. 2018; Rebolloso Hernández et al. 2023).

Understanding the accumulation of trace metals, including species-specific uptake and elimination processes, is essential for assessing their ecological impact and developing effective strategies to mitigate their effects. Toxicokinetics is a way of elucidating these processes, providing deeper insights into the mechanisms of species-specific and metal-specific toxicity to better predict their long-term consequences, even at sublethal doses. Monitoring the internal concentration of a chemical over time has revealed significant variability in how different insect species accumulate and eliminate trace metals, depending on both the species and the trace metal (Vijver et al. 2004; Ardestani et al. 2014). For instance, some species like earthworms accumulate cadmium continuously without reaching a steady state (*Enchytraeus crypticus*: Zhang et al. 2022), whereas others, such as ground beetles, show an initial rapid increase followed by a plateau (*Poecilus cupreus*: Kramarz 1999a). The decontamination phase also varies, with cadmium and lead often being eliminated more efficiently, while zinc and copper tend to be retained longer (earthworm *E. crypticus*: Zhang et al. 2022; ground beetle *P. cupreus*: Kramarz 1999a). Clearly, adding new case studies is necessary for a better understanding of this variability.

Several factors have been suggested to influence these uptake and elimination patterns, and consequently metal bioaccumulation (Luoma and Rainbow 2005) including abiotic environmental factors (such as the level of exposure to a pollutant, food quality and availability and soil characteristics), metal chemical form, mode of exposure (for instance through food, air or soil), and the organism’s ability to regulate internal metal concentrations through various physiological species-specific mechanisms (Grześ 2009, 2010a). Insects, and ants in particular, have very efficient metal regulatory physiology, based on three mechanisms: uptake restriction, metal immobilization and elimination. Metal uptake can be restricted by avoiding contaminated habitats and food or by limiting their assimilation through the gut wall (Grześ 2010a). If metals have crossed the gut barrier, they can be immobilized to prevent them from interfering with metabolic processes. This is achieved by binding to metallothioneins or by depositing them in insoluble granules that can be stored in specific tissues and organs (e.g. midgut epithelium, Malpighian tubes, mandibles; Rabitsch 1997). These granules can also be excreted with faeces or by exuviae, which allows individuals to maintain their internal metal concentration stable (Janssen et al. 1991; Spurgeon and Hopkin 1999; Grześ 2010a; Gekière 2025). However, these detoxification strategies are not cost-free, and maintaining efficient metal regulation through such mechanisms requires energy and resources that could otherwise be allocated to growth or reproduction (Castañeda et al. 2009; Jiang et al. 2023; Gul et al. 2023). As a result, such mechanisms are expected to evolve primarily in polluted environments, where their benefits outweigh their energetic and physiological costs.

The level of environmental pollution is usually reflected in the level of contamination of organisms, but the sensitivity of organisms to the pollution may change through acclimation (phenotypic plasticity) or adaptation (genetic) to high concentrations of pollutants (Morgan et al. 2007). This can result in different detoxification strategies or accumulation patterns in response to different levels of environmental pollution. For example, research on centipedes has produced mixed findings: while *Lithobius forficatus* showed no differences in cadmium and lead kinetics between unpolluted and polluted sites near a smelter (Descamps et al. 1996), a closely related species, *Lithobius variegatus*, exhibited habitat-related differences, with individuals from unpolluted sites displaying higher cadmium accumulation rates than those from highly polluted sites (Hopkin and Martin 1984). In contrast, Grześ (2012) found no significant differences in zinc toxicokinetics between *Myrmica rubra* ants from unpolluted and zinc-polluted sites, as internal zinc concentrations remained stable throughout both contamination and decontamination phases. To our knowledge, no other study has assessed the differences in trace metal toxicokinetics between populations from unpolluted and industrially polluted sites, and none between rural and city species or populations.

Ants are important ecosystem engineers (Sanders and van Veen 2011; Borne et al. 2021), and as ground-dwelling central-place foraging insects with sessile nests, they cannot escape the contaminated environment around them. They are also considered reliable bioindicators and useful bioremediators (Khan et al. 2017; Chowdhury et al. 2023). They can regulate their trace metal body concentration quite effectively (Grześ 2009, 2010a, 2012; Grześ and Okrutniak 2016). Understanding the response of ecologically dominant species such as ants to metal contamination is essential to predict and try to limit the impact of trace metals on ecosystems. However, only one study focused on the toxicokinetics of an essential trace metal, zinc, in ants (Grześ 2012). This study revealed extremely efficient zinc regulation in *M. rubra* ants during both contamination and decontamination phases.

Our study aims to compare the kinetics of internal cadmium concentration in the ant *Temnothorax nylanderi* between two differently polluted habitats with distinct pollution concentrations (forest vs. city) under controlled laboratory conditions during a contamination followed by a decontamination phase. Cadmium, a non-essential element and highly toxic metal, is mostly found in city areas due to various industrial activities, traffic emissions, and improper waste disposal (Alloway 2013; Foti et al. 2017). Its long biological half-life (more than 20 years in humans: Satarug et al. 2003) causes it to accumulate in the body throughout an organism’s lifespan. In social insects, it disrupts essential biological processes across multiple levels, causing reduced energy metabolism, muscle damage, oxidative stress, and weakened immunity, which can harm entire colonies (ant *F. aquilonia*: Migula et al. 1993, 1997; bumblebee *Bombus terrestris*: Gao et al. 2024; bee *A. cerana cerana*: Li et al. 2024; honey bee *A. mellifera*: Polykretis et al. 2016). In the ant *T. nylanderi*, cadmium decreased both adult survival and size of newly emerging adults (Jacquier et al. 2021). Moreover, ants are reliable bioindicators of cadmium pollution, as their level of contamination generally increases with the degree of pollution in their habitats, even though this relationship may sometimes be masked by tolerance mechanisms (Rabitsch 1995a; Grześ 2010b). Interestingly, city colonies of this species did not differ from forest colonies in internal cadmium concentrations despite living in sites with more polluted soil (Gressler et al. 2025). Moreover, they were less negatively impacted by cadmium than their forest counterparts (Jacquier et al. 2021). These results suggest that city colonies might have lower uptake and/or more efficient elimination of cadmium than forest ones. We tested this hypothesis by comparing toxicokinetics between colonies from unpolluted (forest) and polluted (city) sites in two different regions.

## Materials and methods

### Biological model

**Fig. 1:**
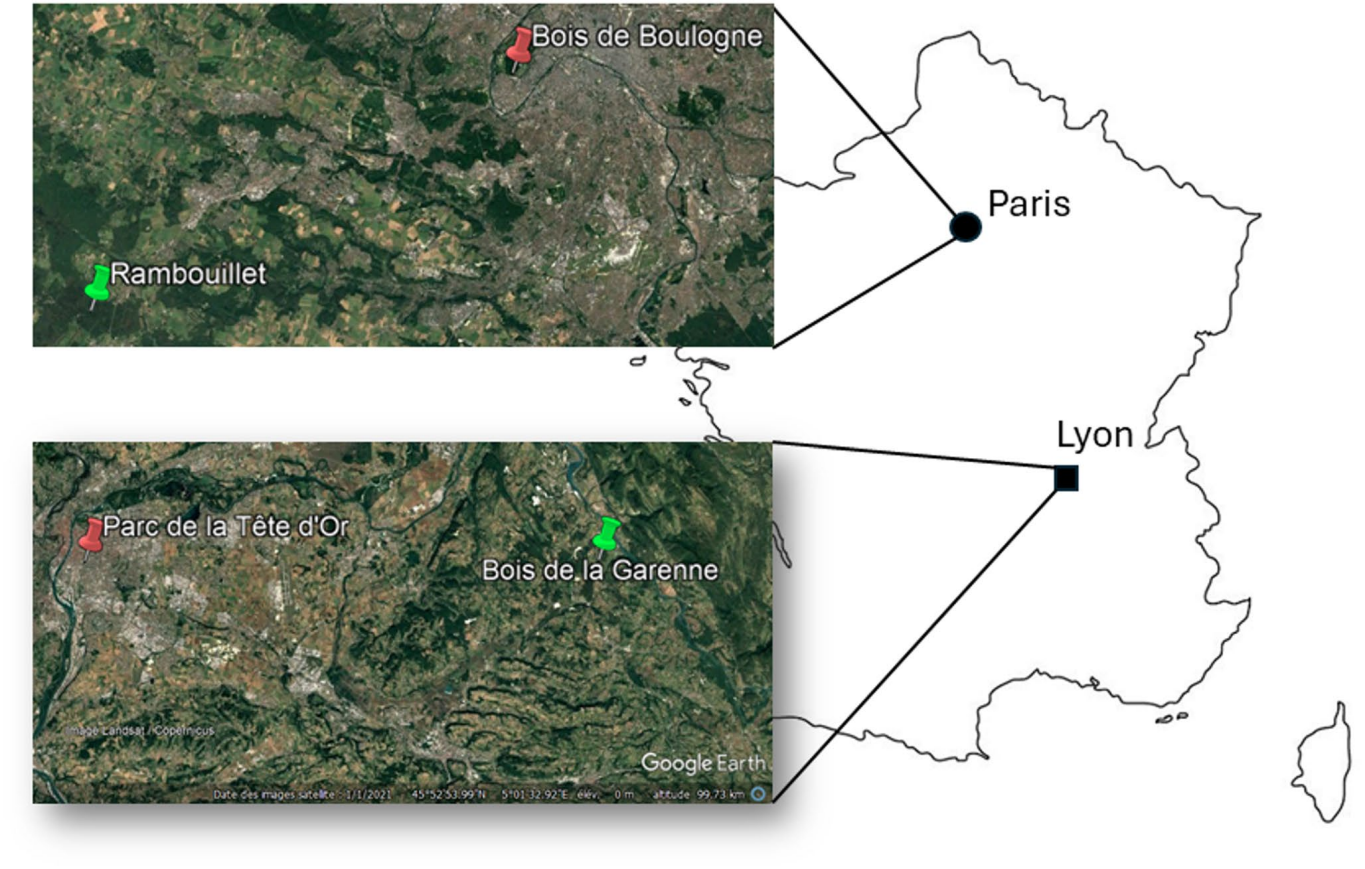
Map of sample locations. Two habitat types were sampled: forest (green) and city park (red). Sampling was conducted in two regions, around Paris and around Lyon. GPS coordinates are provided in Table 1

We used the tiny acorn ant *T. nylanderi* (Foerster 1850) (Formicidae, Myrmicinae, Crematogastrini), which occurs in various habitats in Western Europe (Pusch et al. 2006), including city parks. It forms small colonies (a few dozen to a few hundred individuals; Foitzik and Heinze 1998), with nests in hollow twigs or acorns on the ground, and workers forage less than a meter away (Heinze et al. 2010). In March 2024, we collected colonies from one city park and one forest outside the city in each of the two regions: the Paris region and the Lyon region (four different sites in total; Table 1; Fig. 1). To ensure that the forest sites were minimally contaminated with metals, collections in the forests were carried out away from roads, buildings, and cultivated lands. A previous study found a higher concentration of cadmium in the soils of city parks than in forests in the Paris region, including the two sites sampled here (Gressler et al. 2025). Moreover, higher cadmium concentrations in mosses were also observed in cities compared to rural areas, both in Lyon and Paris (Vieille et al. 2021).

**Table 1:** Sampling characteristics

Region	Habitat	Site & GPS	*N*. Col.	*N*. Col. Discarded				*N*. Col. Exp.	
				QC	IC	SC			
Paris	Forest	Forêt de Rambouillet 48°40’33.9"N 1°48’55.7"E	50	12	1	10	27		
	City	Bois de Boulogne 48°50’57.5"N 2°14’57.0"E	57	18	1	9	29		
Lyon	Forest	Bois de la Garenne 45°44’41.5"N 5°27’17.1"E	54	8	10	2	34		
	City	Parc de la Tête d’Or 45°46’44.4"N 4°51’17.7"E	56	14	0	7	35		

For each site, the region, the habitat types, the location and the number of colonies collected (N. Col) are given. We also report the number of discarded colonies per site (N. Col. Discarded) with the reason for their removal (QC = queenless colonies; IC = infested colonies; SC = small colonies). The final number of colonies used in the experiment is given (N. Col. Exp.)

Colonies were collected in acorns or small twigs, which were opened in the field and placed into small plastic bags when they contained *Temnothorax* ants. The species identification was confirmed in the laboratory by observation under the binocular microscope, according to the identification key of the *Temnothorax nylanderi* species group (Csősz et al. 2015). We collected 104 colonies from forests (50 from the Paris region and 54 from the Lyon region), and 113 from city parks (57 from the Paris region and 56 from the Lyon region; Table 1 and S1). Colonies were housed in the laboratory in artificial nests consisting of two microscope slides separated by a 2 mm layer of black plastic foam, shaped into three chambers, with a sheet of dark paper placed over the nest to ensure darkness. Each nest was placed in a 11.5 × 11.5 × 5.5 cm plastic box serving as a foraging area. We discarded 52 queenless colonies (20 forest colonies and 32 city colonies; Table 1 and S1), 12 queenright colonies that were parasitized (11 forest colonies and one city colony, identifiable by the yellow coloration of cestode-infected workers: Scharf et al. 2012), and 28 colonies that were too small (< 40 workers, 12 forest colonies and 16 city colonies). Among the 125 remaining colonies, we kept only 104 colonies: 52 colonies from forest habitats and 52 colonies from city habitats (Table 1 and S1) to properly balance colony sizes between habitat types and across time points in our experimental design (see below). For the 104 colonies used in the experiment, colony size ranged from 46 to 380 workers for the forest habitats, and from 87 to 438 workers for the city habitats; mean colony size did not differ significantly between the two habitat types at the beginning of our experiment (Table S1). Colonies were kept in a climatic chamber (12 h at 18 °C, 12 h at 22 °C) with *ad libitum* water (tube clogged with cotton placed in the foraging area). Colonies were fed with an ant diet prepared by mixing 340 mL of tap water with 59.143 g of egg, 1.266 g of vitamins (Arkopharma AZINC Vitalité), 3.24 g of agar-agar, and 44.23 g of honey. Colonies were acclimated for one week in the laboratory before the experiment began.

### Experimental design

Colonies from the two regions and the two habitat types were experimentally exposed to two successive feeding treatments. First, for the contamination phase, colonies were fed for 21 days with the ant diet laced with cadmium (a cadmium chloride (CdCl2) solution at a concentration of 49 g/L) to reach the concentration of 100 mg of cadmium per kg of food. This concentration was known to have adverse effects on this species causing, on average, 40% mortality of adult workers after two months of cadmium feeding (Jacquier et al. 2021). A three-week duration of the contamination phase allowed us to avoid high worker mortality (Fig. S1). Second, in the decontamination phase, the colonies were fed for 21 additional days with the same mixture but without cadmium. The durations of both phases were chosen in accordance with similar studies (Janssen et al. 1991; Arini et al. 2013; Bednarska et al. 2015).

Overall, the experiment lasted for 42 days. In the climatic chamber, colonies were randomly distributed between the five racks independently of their locality of origin and the time they were killed for cadmium concentration assessment (time point, see below). Measuring cadmium concentration required 0.025 mg of ants (about 100 individuals), which implied killing the entire colony. Therefore, the points of the toxicokinetic curves were based on different colonies. The internal cadmium concentration of ants was assessed once before the start of the contamination phase (first time point; initial values) and at twelve time points during the experiment (contamination phase at 1, 3, 5, 10, 15, and 21 days; decontamination phase at 22, 24, 26, 31, 36, and 42 days). At each time point, eight colonies (two colonies from the four combinations of habitat types (forest/city) and region (Paris/Lyon)) were taken out of the experiment to measure their internal cadmium concentration. For this purpose, the eight colonies were starved for 48 h to empty the workers’ guts before being frost-killed. Dead workers of each colony were counted and removed from the sample so that only alive workers were analyzed for cadmium concentration. Three forest colonies (RAM44, RAM10, and RAM20, Table S1) had a higher daily worker mortality rate right after starting the experiment than the other colonies (between 5% and 10% at days 1 and 3 versus < 2% for the other colonies at the same time points; Fig. S1). We therefore decided to remove these three colonies from the subsequent statistical analyses. Note that removing these three colonies did not affect the qualitative conclusion of the statistical analyses.

### Chemical analyzes

We dried the frozen colonies in an oven at 40 °C for 48 h and then sent them to the FiLAB laboratory (https://filab.fr/en/) for mineralization and analysis of cadmium concentration using Inductively Coupled Plasma Mass Spectrometry (ICP-MS). All colonies were weighed without washing the ants before the mineralization. For mineralization, a multi-acid reaction mixture (nitric acid HNO_3_ (2 ml) and hydrofluoric acid HF (1 ml)) was added to each sample, followed by a heating step (two hours at 100 °C) performed on a heating block. After returning to ambient temperature, ultra-pure water was added to each sample to reach a final volume of 10 mL. An aliquot of each sample solution was taken for ICP-MS analysis. The ICP-MS analyzer (Brand THERMO FISHER model ICAP RQ) was calibrated using certified solutions over a range of 10 µg/L to 20 µg/L. Quality Controls (QCs: synthetic points carried out independently of the calibration range to check that the analytical sequence does not drift) and Reference Materials (RMs: certified standards at a known concentration which are run through the analytical series and enable to validate the calibration ranges) were inserted throughout the series to check the range accuracy, measurement sensitivity and absence of drift (throughout the analytical sequence, the internal standard percentages ranged from 72% to 114%). These QCs were prepared at strategic concentrations: limit of quantification, mid-range and high-range. Several verifications were then carried out during reprocessing of the results: checking the range data and various QC/RMs, and verification of the specificity of the measurement by interpretation of the different isotopes of the element Cd. Finally, the analytical series were validated in accordance with the requirements of the FiLAB laboratory. For each colony, FiLAB thus provided us with a cadmium concentration in mg∙kg^− 1^ of ant dry tissue.

### Statistical analyses and modelling

All statistical analyses were carried out using R v.4.2.2 (https://www.r-project.org/), and all plots were generated using the *ggplot2* package (Wickham 2009).

The pattern of the internal cadmium concentration in ant colonies (*C*_*I*_ [mg∙kg^− 1^]) over time (*t* [days]) was described using the one-compartment model from Skip et al. (2014):

for the contamination phase (*t* ≤ *t*_*C*_):

1 $$\begin{array}{lll}{C}_{I}\left(t\right)={C}_{I0}\cdot\:{e}^{-{k}_{E1}\cdot\:t}\\\qquad\qquad+\,{C}_{Eu}\cdot\:\frac{{k}_{A1}}{{k}_{E1}}\cdot\:\left(1-{e}^{-{k}_{E1}\cdot\:t}\right)\end{array}$$

and for the decontamination phase (*t* > *t*_*C*_):

2 $$\begin{array}{lll}{C}_{I}\left(t\right)={C}_{I{t}_{C}}\cdot\:{e}^{-{k}_{E2}\cdot\:(t-{t}_{C})}\\\qquad\qquad+\,{C}_{Ed}\cdot\:\frac{{k}_{A2}}{{k}_{E2}}\cdot\:\left(1-{e}^{-{k}_{E2}\cdot\:(t-{t}_{C})}\right)\end{array}$$

where

3 $$\begin{array}{lll}{C}_{I{t}_{C}}={C}_{I0}\cdot\:{e}^{-{k}_{E1}\cdot\:{t}_{C}}\\\qquad\qquad+\,{C}_{Eu}\cdot\:\frac{{k}_{A1}}{{k}_{E1}}\cdot\:\left(1-{e}^{-{k}_{E1}\cdot\:{t}_{C}}\right)\end{array}$$

where *C*_*I0*_ is the internal cadmium concentration in the ants at the start of the uptake phase (initial measures, at *t* = 0; [mg∙kg^− 1^]), and *C*_*E*_ is the external cadmium concentration in the food [mg∙kg^− 1^] during the contamination phase (*C*_*Eu*_) and the decontamination phase (*C*_*Ed*_); *k* is the kinetic parameters [day^− 1^] estimated by the model: assimilation rate constant (*k*_*A*_) and elimination rate constant (*k*_*E*_); and *t*_*C*_ is the time of changing the food from contaminated to uncontaminated [days]. Both parts of the model (*Eqs. 1 and 2)* were fitted separately for each phase, allowing the kinetic parameters to be different between the two phases (*k*_*A1*_ and *k*_*E1*_, *k*_*A2*_ and *k*_*E2*_). Least squares fitting to estimate the kinetic parameters was done using the Gauss-Newton algorithm (*nls* function from the *stats* package). The initial internal concentration at the beginning of the contamination phase *C*_*I0*_ was given in the model as the average internal cadmium concentration measured in the eight initial colonies. Cadmium concentrations in the food, both *C*_*Eu*_ and *C*_*Ed*_, were not explicitly measured for this experiment, and were given in the model as *C*_*Eu*_ = 100 mg∙kg^− 1^ and *C*_*Ed*_ = 1 mg∙kg^− 1^. The time of switching from the contamination phase to the decontamination phase *t*_*C*_ was 21 days. The internal concentration at the beginning of the decontamination phase *C*_*ItC*_ (*Eq. 3*) was derived from the predicted value at *t = t*_*C*_ using the equation for the contamination phase (*Eq. 1*) and used as the starting value for the equation of the decontamination phase (*Eq. 2*), ensuring continuity between the two phases. To compare kinetics between habitat types (forest vs. city) for each phase separately (contamination vs. decontamination), a model allowing kinetic parameters to differ between habitat types (habitat model) was compared to a model estimating the kinetic parameters on the entire dataset (both habitat types at once, null model) for each phase separately. We first compared these models using the AIC values. When the habitat model had a lower AIC value, explaining the data better, we used the *F*-test (*anova* function from the *stats* package) to get a significance value.

To assess whether the internal cadmium concentration at the end of the decontamination phase was different from the initial concentration, we used a linear model (*lm* function, package *stats*) with the internal cadmium concentration in ant colonies as dependent variable, the time point (*t* = 0 vs. *t* = 42), the habitat type, and their first order interaction as fixed factors: *Internal Cd concentration ~ Time point + Habitat + Time point : Habitat*. The *F* tests and *P*-values assessing the effect of the factor of interest were obtained by comparing the full model with a model excluding the factor of interest. The normality of residuals and homogeneity of variances were visually checked according to Pinheiro and Bates (2000), and no transformation of the data was required.

### Ethical note

No ethical oversight is required to work with insects in France. However, we maintained the animals under non-stressful conditions and reproduced the field environmental conditions (temperature) required for optimal rearing. Workers were frost-killed.

## Results

### General toxicokinetics

**Fig. 2:**
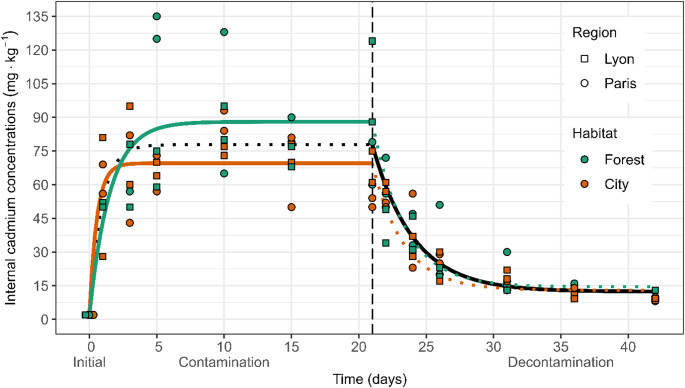
Internal cadmium concentration (mg∙kg^− 1^) and associated toxicokinetic curves. Dot colours correspond to habitat types: forest in green, city in orange. Dot shapes correspond to sampling regions: squares for the Lyon region, circles for the Paris region. The lines represent the fit to the data of the toxicokinetic models: overall model in black, model indexed by habitat type in green (forest) and orange (city); the plain lines represent the model that best explains the data for each phase (the coloured ones for the first phase and the black one for the second phase). The broken vertical line represents the time when colonies were switched from the contamination phase to the decontamination phase (*t*_*C*_ = 21 days). Horizontal jitter was applied for the eight data points at *t* = 0 (initial, after field collection) to enhance readability as they all had values around 2 mg∙kg^− 1^

When colonies were fed with the contaminated food (contamination phase), internal cadmium concentration increased rapidly over the first three days, followed by a plateau from the fifth to the 21st day and even slightly decreasing at the end of the contamination phase (Fig. 2). When switching to uncontaminated food at day 21 (decontamination phase), internal cadmium concentration decreased rapidly over about 10 days and seemed to reach a steady-state level at the end of the experiment with low internal cadmium concentrations (Fig. 2). Colonies showed greater variability in internal cadmium concentrations during the contamination phase than during the decontamination phase, with especially high values over 120 mg∙kg^− 1^ for four of the forest colonies (colonies 27, 28, 36, and 54 at *t* = 5, 10 and 21; Fig. 2, Table S1) compared to the others that never exceeded the cadmium concentration in food of 100 mg∙kg^− 1^. These four colonies were within the normal size range, being neither unusually small nor unusually large.

### Toxicokinetic differences between habitat types

Initially, internal cadmium concentration in ants was low and did not differ between forest and city habitats (all colonies, regardless of their habitat type, were < 2 mg∙kg^− 1^, Fig. 2). In the contamination phase, the model with toxicokinetic parameters fitted by habitat type explained the data better than the null model (AIC_null_ = 469.53; AIC_habitat_ = 464.87; *F*_2,51_ = 4.35, *p* = 0.018), indicating a significant difference in the internal cadmium concentration kinetics between the forest and the city habitats (Fig. 2). City colonies showed a slightly faster initial increase in internal cadmium concentration compared to forest colonies but had a plateau at a lower concentration than forest colonies (88 and 69.55 mg∙kg^− 1^ for the forest and city colonies respectively; Fig. 2., Table 2). In the decontamination phase, the model with toxicokinetic parameters fitted by habitat types was inferior to the null model in explaining our data (AIC_null_ = 438.84; AIC_habitat_ = 456.46; Fig. 2; Table 2*).* Fitting different parameters by habitat types therefore did not add significant information. The internal cadmium concentration was significantly higher at the end of the experiment than before starting the contamination phase for both habitat types (*t* = 0: lower than 2 mg∙kg^− 1^; *t* = 42: forest: 5.74 mg∙kg^− 1^, city: 5.66 mg∙kg^− 1^; comparison between *t* = 0 and *t* = 42: *F*_1,14_ = 160.72, *p* < 0.001; non-significant interaction with habitat: *F*_1,13_ = 0.01, *p* = 0.90; non-significant effect of habitat: *F*_1,14_ = 0.01, *p* = 0.90), indicating that neither forest nor city colonies returned to their initial internal cadmium concentration at the end of the decontamination phase.

**Table 2:** Estimated toxicokinetic parameters and associated statistics

Type of model	Habitat	Contamination phase			Decontamination phase			
		k_A1_	k_E1_	*R* ^2^	k_A2_	k_E2_	*R* ^2^	
Null		0.871 0.460–1.282	1.119 0.563–1.674	0.647	4.075 1.805–6.345	0.33 0.248–0.412	0.774	
Habitat	Forest	0.581 0.27–0.892	0.66 0.28–1.041	0.688	6.067 1.541–10.583	0.419 0.266–0.572	0.710	
	City	1.262 0.252–2.272	1.815 0.302–3.327		5.601 1.214–9.988	0.431 0.276–0.585		

The estimated *k*_*A*_ (assimilation rate constant [day^− 1^]) and *k*_*E*_ (elimination rate constant [day^− 1^]) were allowed to differ between the two phases of the experiment (the contamination phase (_1_) and the decontamination phase (_2_)). Ranges correspond to asymptotic 95% confidence intervals. *R*^2^ represents the coefficient of determination of the fitted model of each phase, adjusted for the degrees of freedom. Results are presented for a model without (Null) and with (Habitat) the possibility of fitting different parameters for each habitat type. Note that the estimates were obtained assuming values of *C*_*Eu*_ = 100 mg·kg⁻¹ and *C*_*Ed*_ = 1 mg·kg⁻¹ for the contaminated and uncontaminated food, respectively

## Discussion

By comparing the temporal kinetics of internal cadmium concentration in the ant *T. nylanderi* between two habitat types with contrasting levels of pollution (forest vs. city), we observed that city colonies reached a lower plateau than forest colonies during the contamination phase, whereas no significant difference was detected during the decontamination phase. Overall, both habitat types exhibited a similar toxicokinetic pattern: cadmium concentration increased rapidly at the onset of the contamination phase, stabilized at a plateau, and then declined sharply during the decontamination phase.

*T. nylanderi* ants accumulated cadmium in their body during the contamination phase, which is consistent with other studies on ants regarding cadmium, zinc, lead, and copper (Rabitsch 1995b; Grześ 2010a). The kinetics of cadmium accumulation with a fast increase followed by a plateau was also found for other soil-dwelling organisms (centipede *L. variegatus*: Hopkin and Martin 1984; cricket *Gryllus assimilis*: Bednarska et al. 2015; ground beetle *P. cupreus*: Kramarz 1999a). The fact that a plateau was reached after only a few days of cadmium contaminated diet, and that the concentration at the plateau was not higher than the concentration of the contaminated food (77.84 mg∙kg^− 1^ for both habitat types vs. expected 100 mg∙kg^− 1^ for the food), indicates that *T. nylanderi* ants are capable of regulating internal cadmium concentration, which is consistent with the findings of Grześ (2009) on cadmium regulation in *Lasius niger* ants. An exact quantification of the cadmium concentration in the food and of the quantity of food taken by ants would be necessary to confirm this result. However, in some cases, this regulatory capacity may be surpassed, as four forest colonies exceeded the cadmium concentration in food, reaching levels above 120 mg∙kg⁻¹. This suggests that even with efficient metal regulation mechanisms, bioaccumulation can lead to internal cadmium concentrations surpassing environmental concentrations. A similar phenomenon was observed by Grześ (2012), where zinc concentration in one *M. rubra* colony exceeded the concentration in contaminated food. Additionally, comparable cases of cadmium bioaccumulation beyond food or substrate concentration have been documented in oribatid mites (*Platynothrus peltifer*: Janssen et al. 1991; Janssen and Bergema 1991) and pseudoscorpions (*Neobisium muscorum*: Janssen et al. 1991), as well as in enchytraeids (*E. crypticus*), which accumulated higher cadmium concentrations than those present in contaminated soils (Zhang et al. 2022).

Once the contaminated food was replaced with uncontaminated food in our experiment, a fast decrease in internal cadmium concentrations occurred, indicating efficient elimination of cadmium in *T. nylanderi* ants. This detoxification dynamics was similar to other soil-dwelling invertebrates regarding cadmium (ground beetle *P. cupreus*: Kramarz 1999a; centipede *Lithobius mutabilis*: Kramarz 1999b; collembolan *Orchesella cincta*: Janssen et al. 1991; Janssen and Bergema 1991; carabid *Notiophilus biguttatus*: Janssen et al. 1991). Ants, and insects in general, are known for their ability to regulate internal metal concentration, leading to an increased tolerance (Grześ 2012; Khan et al. 2017). Several detoxification pathways exist, like the use of metallothioneins, insoluble granules, or reduced cadmium uptake at the gut level (Janssens et al. 2009; Roelofs et al. 2009; Grześ 2010b; Ardestani et al. 2014).

At the end of the decontamination phase, internal cadmium concentrations in ants returned to a steady state at low concentrations, though they remained significantly higher than the initial internal cadmium concentrations. Similar results were obtained regarding cadmium in collembolans (*O. cincta*: Janssen et al. 1991; Janssen and Bergema 1991) and lead in enchytraeids (*E. crypticus*: Zhang et al. 2022). In contrast, studies with the same toxicokinetic patterns showed that the internal concentrations did return to or near their initial levels at the end of decontamination for cadmium in ground beetles (*P. cupreus*: Kramarz 1999a), centipedes (*L. mutabilis*: Kramarz 1999b) and carabids (*N. biguttatus*: Janssen et al. 1991), as well as for copper in enchytraeids (*E. crypticus*: Santos et al. 2021). This diversity does not appear to result from differences in the duration of the decontamination phase. For instance, studies that reported a return to normal internal concentrations at the end of their experiment had decontamination phases lasting 35 days (Janssen et al. 1991), 31 days (Kramarz 1999a, b), and 14 days (Santos et al. 2021), while those that did not find a return to normal internal concentrations lasted 56 days (Janssen and Bergema 1991), 30 days (Janssen et al. 1991), and 14 days (Zhang et al. 2022). Our decontamination phase lasted 21 days, and a longer decontamination phase would be necessary to know whether *T. nylanderi* is able to return to its initial state after being contaminated. If not, this would suggest that prolonged or extreme exposure to pollutants alters the detoxification mechanisms or that contaminants are immobilized in insoluble granules without being excreted (Crommentuijn et al. 1994; Grześ 2010b; Gekière 2025). This could have ecological implications such as bioaccumulation along food webs and long-term disturbances of ecosystem services.

Interestingly, we found that city *T. nylanderi* colonies had a lower plateau than forest colonies during the contamination phase. This suggests that city colonies regulate their internal cadmium concentration better, which could explain why city *T. nylanderi* ants were more tolerant to cadmium than their forest counterparts, as found by Jacquier et al. (2021). It also corroborates findings on other insects where populations from heavily metal-polluted sites have higher tolerance to trace metals (centipede *L. variegatus*: Hopkin and Martin 1984; grasshopper *Chorthippus brunneus*: Augustyniak and Migula 2000). The lack of difference in cadmium elimination between ant colonies from the two habitat types during the decontamination phase suggests that forest colonies are just as efficient as city colonies in removing cadmium. Forest colonies began the decontamination phase with higher cadmium concentrations than city colonies but reached similar levels by the end. Therefore, the difference observed in the contamination phase may result from variations in cadmium uptake or assimilation rather than from differences in elimination capacities. This could for instance involve differences in metal assimilation through the gut wall or in the capacity to detect and avoid contaminated food before ingestion. It is possible that city colonies consumed less cadmium-contaminated food by choosing to starve instead of risking contamination. Similar behaviours have been observed in grasshoppers, which can select unpolluted food (*Chorthippus* sp.: Migula and Binkowska 1993), and in bees, which avoid contaminated food when concentrations are sufficiently high (*A. mellifera*: Monchanin et al. 2022). However, we cannot confirm these hypotheses in this current study, as neither colony weight nor food consumption was monitored throughout the experiment.

Our findings suggest that chronic cadmium exposure in city habitats might contribute to differences in cadmium regulation by *T. nylanderi* colonies. Whether this difference could be genetically determined remains to be investigated. Forest and city populations of *T. nylanderi* of these two regions did not genetically differ except for a limited number of single-nucleotide polymorphisms (SNPs) (three for the Paris region and eight for the Lyon region; Khimoun et al. 2020). However, none of the identified loci were linked to known detoxification or stress-response pathways. More investigation should be carried out to assess the genetic and/or plastic origins of the difference observed.

Urban environments present a combination of stressors, including chemical pollutants (Barinova et al. 2020), light (Gaston et al. 2012) and noise (Barber et al. 2010), and elevated temperatures (Kim 1992), which could create diverse selection pressures and amplify physiological responses. For instance, *Gammarus pulex* exhibited stronger adverse effects when exposed to a pesticide combined with temperature stress (Russo et al. 2018), and mixtures of trace metals elicited more pronounced stress responses than single metals (*D. melanogaster*: Frat et al. 2021). These findings underscore the need to consider the interactive effects of multiple sources of physiological stress when studying the physiological responses of urban organisms. Such synergistic effects may also occur in *T. nylanderi*, where city colonies are exposed to various pollutants, including several heavy metals. As such, the response we observed in city colonies may not have evolved specifically as a direct adaptation to cadmium contamination. Instead, it may represent a by-product of selection pressures exerted by other stressors. Notably, Gressler et al. (2025) found that city colonies of *T. nylanderi* had higher lead concentrations than forest colonies, whereas levels of cadmium, copper, and zinc did not differ between colonies from the two habitat types. This raises the possibility that lead pollution could act as a priming factor for detoxification mechanisms, potentially enhancing the efficiency of cadmium regulation in city colonies. Future studies manipulating both cadmium exposure and additional stressors (such as other chemical pollutants, temperature, or parasites) would help clarify how urban stressors collectively influence toxicokinetics and trace metal handling in ants. This approach would also help determine whether the observed responses in city colonies are cadmium-specific or reflect a broader physiological adaptation to the complex selection pressures of urban environments.

## Conclusions

Our results revealed distinct cadmium toxicokinetics in *T. nylanderi* ant colonies from forest and city environments, with city colonies showing a lower internal concentration plateau when fed a cadmium-contaminated diet. This difference suggests an enhanced ability of city colonies to limit cadmium assimilation, likely contributing to their previously observed elevated resistance to cadmium (Jacquier et al. 2021). The contamination phase may be critical in explaining the higher resistance of ants inhabiting cities to pollution. It remains to be clarified whether the observed resistance is due to phenotypic plasticity or specific genetically fixed physiological adaptations. Ultimately, these insights could reveal whether ants can serve as bioindicators for monitoring trace metal pollution, or as bioremediators if they limit the transfer of pollutants into higher levels of the trophic network.

## Supplementary Information

Below is the link to the electronic supplementary material.

Supplementary Material 1

## Data Availability

Our dataset is available on Zenodo: [https://doi.org/10.5281/zenodo.15267788](https:/doi.org/10.5281/zenodo.15267788) .
