# Supplementary material for: Cadmium toxicokinetics differ between forest and city colonies of the ant Temnothorax nylanderi (Formicidae: Myrmicinae) (Foerster, 1850)
Source: Ecotoxicology. 2026 Feb 19;35(3):63. doi: 10.1007/s10646-026-03053-4 (PMC12920765; doi:10.1007/s10646-026-03053-4)
Supplement: Supplementary file 1 — Supplementary Material 1 [file 10646_2026_3053_MOESM1_ESM.docx]

**SUPPLEMENTARY MATERIALS**

Cadmium toxicokinetics differ between forest and city colonies of the ant *Temnothorax nylanderi* (Formicidae: Myrmicinae) (Foerster, 1850)

Marie Gressler ^a^, Angélique Bultelle ^a^, Bernard Kaufmann^d^, Mathieu Molet ^a*^ & Claudie Doums ^bc*^

^a^ Sorbonne Université, UPEC, CNRS, IRD, INRA, Institute of Ecology and Environmental Sciences (IEES-Paris); 4 place Jussieu, 75005 Paris, France; angelique.bultelle@ird.fr; mathieu.molet@sorbonne-universite.fr

^b^ Institut de Systématique, Evolution, Biodiversité (ISYEB), EPHE-PSL, Muséum National D'Histoire Naturelle, CNRS, Sorbonne Université, Université des Antilles; 57 rue Cuvier, CP 50, 75005 Paris, France

^c^ EPHE, PSL University

^d^ Université Claude Bernard Lyon 1, LEHNA UMR 5023, CNRS, ENTPE, F-69622, Villeurbanne, France

* co-last authors

Corresponding author: Claudie Doums ; claudie.doums@ephe.psl.eu; ISYEB, 57 rue Cuvier, CP 50, 75005 Paris, France

**Table S1.** Colony characteristics. All colonies collected in the field are presented with their habitat type, region (Lyon or Paris) and their colony size. Colonies with a sample number were used in the experiment. For these colonies, we added the time point, the time at which the colony was killed and the cadmium concentration estimated. For all 125 field colonies, colony size ranged from 46 to 380 workers for the forest habitat, and from 85 to 438 for the city habitat; colony size did not differ significantly between habitats (Wilcoxon nonparametric test; *W* = 1941.5, *P* = 0.961). For the 104 colonies used in the experiment (52 forest colonies and 52 city colonies), colony size ranged from 46 to 380 workers for the forest habitat, and from 87 to 438 for the city habitat; colony size did not differ significantly between habitats (Wilcoxon nonparametric test*; W* = 1233.5, *P* = 0.443).

| Colony ID | Habitat | Region | Colony size | Sample number | Time point |
| --- | --- | --- | --- | --- | --- |
| ARA1 | Forest | Lyon | 109 | 7 | 0 |
| ARA10 | Forest | Lyon | 208 | 78 | 26 |
| ARA11 | Forest | Lyon | 216 | 70 | 24 |
| ARA13 | Forest | Lyon | 152 | 40 | 10 |
| ARA14 | Forest | Lyon | 312 | 48 | 15 |
| ARA16 | Forest | Lyon | 223 | . | . |
| ARA17 | Forest | Lyon | 112 | 94 | 36 |
| ARA18 | Forest | Lyon | 139 | 62 | 22 |
| ARA21 | Forest | Lyon | 195 | . | . |
| ARA22 | Forest | Lyon | 122 | 31 | 5 |
| ARA28 | Forest | Lyon | 169 | . | . |
| ARA3 | Forest | Lyon | 90 | . | . |
| ARA31 | Forest | Lyon | 270 | 54 | 21 |
| ARA32 | Forest | Lyon | 151 | 85 | 31 |
| ARA33 | Forest | Lyon | 248 | 14 | 1 |
| ARA38 | Forest | Lyon | 261 | 39 | 10 |
| ARA39 | Forest | Lyon | 134 | 47 | 15 |
| ARA4 | Forest | Lyon | 253 | 93 | 36 |
| ARA42 | Forest | Lyon | 144 | 53 | 21 |
| ARA43 | Forest | Lyon | 170 | . | . |
| ARA45 | Forest | Lyon | 192 | . | . |
| ARA47 | Forest | Lyon | 180 | 69 | 24 |
| ARA48 | Forest | Lyon | 107 | 102 | 42 |
| ARA49 | Forest | Lyon | 240 | 20 | 3 |
| ARA5 | Forest | Lyon | 188 | 101 | 42 |
| ARA50 | Forest | Lyon | 222 | 86 | 31 |
| ARA51 | Forest | Lyon | 247 | 61 | 22 |
| ARA52 | Forest | Lyon | 96 | . | . |
| ARA53 | Forest | Lyon | 107 | 77 | 26 |
| ARA55 | Forest | Lyon | 179 | 32 | 5 |
| ARA56 | Forest | Lyon | 149 | 19 | 3 |
| ARA6 | Forest | Lyon | 380 | 8 | 0 |
| ARA7 | Forest | Lyon | 106 | 13 | 1 |
| ARA9 | Forest | Lyon | 217 | . | . |
| RAM1 | Forest | Paris | 193 | 49 | 21 |
| RAM10 | Forest | Paris | 93 | 12 | 1 |
| RAM18 | Forest | Paris | 176 | 66 | 24 |
| RAM19 | Forest | Paris | 129 | 65 | 24 |
| RAM20 | Forest | Paris | 127 | 24 | 3 |
| RAM21 | Forest | Paris | 95 | 5 | 0 |
| RAM22 | Forest | Paris | 117 | 81 | 31 |
| RAM23 | Forest | Paris | 129 | 28 | 5 |
| RAM24 | Forest | Paris | 180 | 50 | 21 |
| RAM26 | Forest | Paris | 262 | 6 | 0 |
| RAM28 | Forest | Paris | 161 | 58 | 22 |
| RAM30 | Forest | Paris | 139 | 41 | 15 |
| RAM33 | Forest | Paris | 46 | 42 | 15 |
| RAM35 | Forest | Paris | 131 | 89 | 36 |
| RAM39 | Forest | Paris | 91 | 27 | 5 |
| RAM40 | Forest | Paris | 122 | 57 | 22 |
| RAM41 | Forest | Paris | 86 | . | . |
| RAM43 | Forest | Paris | 284 | 73 | 26 |
| RAM44 | Forest | Paris | 173 | 11 | 1 |
| RAM45 | Forest | Paris | 315 | 23 | 3 |
| RAM47 | Forest | Paris | 198 | 35 | 10 |
| RAM49 | Forest | Paris | 105 | 90 | 36 |
| RAM5 | Forest | Paris | 203 | 74 | 26 |
| RAM51 | Forest | Paris | 189 | 82 | 31 |
| RAM54 | Forest | Paris | 157 | 36 | 10 |
| RAM6 | Forest | Paris | 240 | 97 | 42 |
| RAM9 | Forest | Paris | 125 | 98 | 42 |
| TOR10 | City | Lyon | 97 | 63 | 22 |
| TOR11 | City | Lyon | 143 | 37 | 10 |
| TOR13 | City | Lyon | 173 | 17 | 3 |
| TOR15 | City | Lyon | 151 | 71 | 24 |
| TOR16 | City | Lyon | 169 | . | . |
| TOR17 | City | Lyon | 296 | 2 | 0 |
| TOR19 | City | Lyon | 170 | . | . |
| TOR20 | City | Lyon | 94 | 1 | 0 |
| TOR21 | City | Lyon | 191 | . | . |
| TOR22 | City | Lyon | 196 | 95 | 36 |
| TOR24 | City | Lyon | 170 | 64 | 22 |
| TOR25 | City | Lyon | 100 | . | . |
| TOR27 | City | Lyon | 302 | 45 | 15 |
| TOR29 | City | Lyon | 106 | 55 | 21 |
| TOR3 | City | Lyon | 160 | 88 | 31 |
| TOR31 | City | Lyon | 109 | 18 | 3 |
| TOR34 | City | Lyon | 161 | 80 | 26 |
| TOR35 | City | Lyon | 158 | 103 | 42 |
| TOR37 | City | Lyon | 126 | 96 | 36 |
| TOR38 | City | Lyon | 120 | 38 | 10 |
| TOR39 | City | Lyon | 117 | 29 | 5 |
| TOR40 | City | Lyon | 126 | . | . |
| TOR42 | City | Lyon | 164 | . | . |
| TOR43 | City | Lyon | 110 | 87 | 31 |
| TOR44 | City | Lyon | 195 | 30 | 5 |
| TOR47 | City | Lyon | 249 | 56 | 21 |
| TOR48 | City | Lyon | 163 | 79 | 26 |
| TOR5 | City | Lyon | 85 | . | . |
| TOR50 | City | Lyon | 126 | . | . |
| TOR51 | City | Lyon | 154 | 46 | 15 |
| TOR54 | City | Lyon | 263 | 16 | 1 |
| TOR55 | City | Lyon | 215 | 72 | 24 |
| TOR56 | City | Lyon | 162 | . | . |
| TOR57 | City | Lyon | 220 | 104 | 42 |
| TOR7 | City | Lyon | 130 | 15 | 1 |
| BOU11 | City | Paris | 155 | 33 | 10 |
| BOU12 | City | Paris | 99 | . | . |
| BOU13 | City | Paris | 188 | 52 | 21 |
| BOU14 | City | Paris | 215 | 10 | 1 |
| BOU15 | City | Paris | 377 | 21 | 3 |
| BOU16 | City | Paris | 101 | . | . |
| BOU17 | City | Paris | 237 | 44 | 15 |
| BOU18 | City | Paris | 285 | 26 | 5 |
| BOU2 | City | Paris | 121 | 9 | 1 |
| BOU23 | City | Paris | 85 | . | . |
| BOU24 | City | Paris | 107 | 51 | 21 |
| BOU25 | City | Paris | 164 | 100 | 42 |
| BOU27 | City | Paris | 87 | 22 | 3 |
| BOU28 | City | Paris | 253 | 34 | 10 |
| BOU29 | City | Paris | 205 | 76 | 26 |
| BOU31 | City | Paris | 257 | 91 | 36 |
| BOU32 | City | Paris | 117 | 59 | 22 |
| BOU34 | City | Paris | 159 | 67 | 24 |
| BOU37 | City | Paris | 275 | 60 | 22 |
| BOU39 | City | Paris | 236 | 92 | 36 |
| BOU44 | City | Paris | 438 | 4 | 0 |
| BOU45 | City | Paris | 221 | 84 | 31 |
| BOU47 | City | Paris | 188 | 75 | 26 |
| BOU5 | City | Paris | 131 | 99 | 42 |
| BOU51 | City | Paris | 322 | 68 | 24 |
| BOU52 | City | Paris | 111 | 3 | 0 |
| BOU6 | City | Paris | 119 | 83 | 31 |
| BOU7 | City | Paris | 164 | 25 | 5 |
| BOU8 | City | Paris | 320 | 43 | 15 |


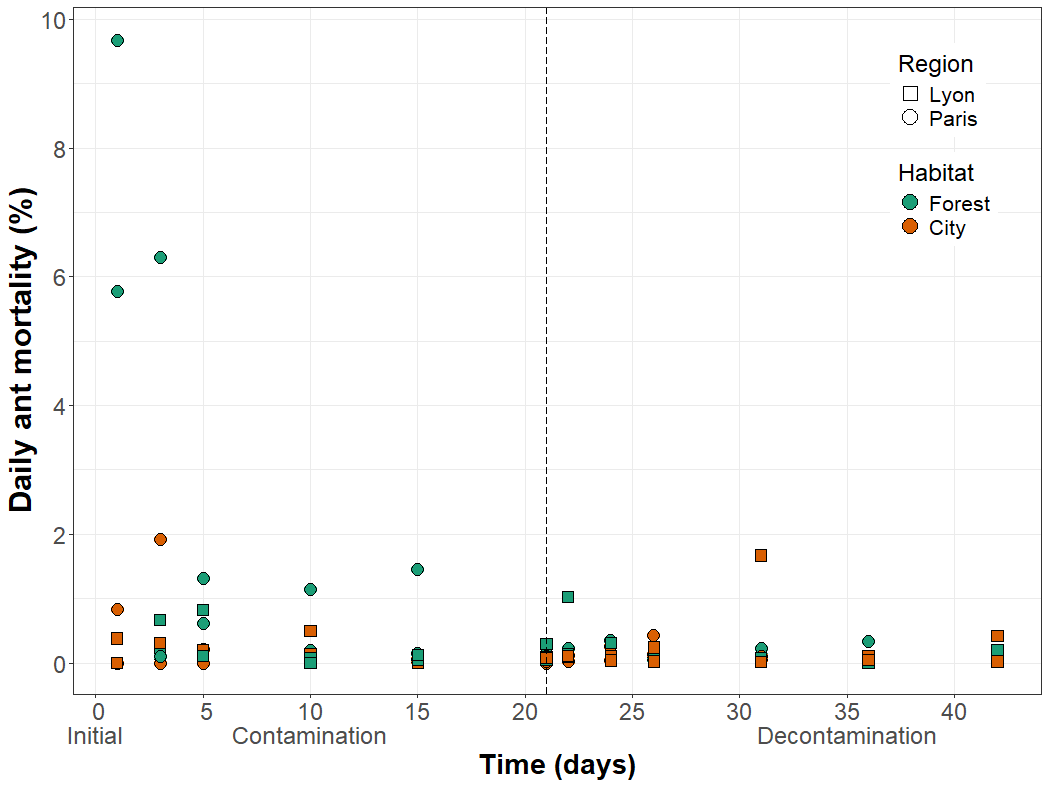
**Figure S1.** Daily ant mortality over the 42 days of the experiment. The daily ant mortality is estimated as the ratio of the percentage of the number of dead workers per initial colony size per time point. Colours correspond to habitat types: forest in green, city in orange. Shapes correspond to sampling region: squares for Lyon region, circles for Paris region. The broken vertical line represents the time from which colonies were switched from contamination phase to decontamination phase (*t_C_* = 21 days).
